# Is personality linked to season of birth?

**DOI:** 10.1371/journal.pone.0253815

**Published:** 2021-06-25

**Authors:** Hoseon Lee, Hye-Kyung Lee, Kounseok Lee

**Affiliations:** 1 Seodaemoonbom Psychiatric Clinic, Seoul, Republic of Korea; 2 Department of Psychiatry, College of Medicine, Hanyang University, Seoul, Republic of Korea; 3 Department of Nursing, College of Nursing and Health, Kongju National University, Gongju, Republic of Korea; Washington University, St. Louis, UNITED STATES

## Abstract

The environment is a very significant factor in early childhood development. Season of birth (SOB) is a proxy viable for the environment to which the babies are exposed, thus also significant in early development. This study investigates the association between SOB and personality. A total 2,962 college students were included as study participants. The participants were classified into four seasonal groups based on their birth month and underwent a personality assessment using the Temperament and Character Inventory (TCI). Statistical analysis was performed using one-way analysis of variance (ANOVA) and multinomial logistic regression analysis. The male participants born in autumn scored high on the Disorderliness (NS4) subscale (β = 0.055, *P* = 0.042) and the male participants born in summer and winter scored high on the Extravagance (NS3) subscale (summer: β = 0.072, *P* = 0.01, winter: β = 0.078, *P* = 0.003). The difference observed indicates a relationship between the SOB and temperament, especially NS. Our findings suggest that environmental factors may affect temperament in early development, although further research is likely needed to clarify the causality between them.

## Introduction

There is a significant association between SOB and personality traits [1–3]. The SOB is often considered when assessing the environmental impacts associated with psychological and psychiatric phenomena [4]. The SOB is a non-specific environmental variable that includes various lifestyles, including latitude, sunshine, nutrition, infection, and stress, among the environmental impacts [5, 6]. Seasonal changes are affected by these environmental variables, and SOB includes many environmental variables [7, 8]. The SOB affects growth and development even in the fetal stage. In the early stages of human development, environmental variables such as SOB, air pollution, dietary habits, lack of vitamin D, maternal infections, and temperature changes may affect growth and development [7, 9, 10].

There is an association between SOB and cerebrospinal fluid (CSF) central monoamine or monoamine metabolites [11–13]. Studies have shown that monoamine function and monoamine turnover affect personality traits [14]. According to a study of adults in Stockholm, Sweden, the SOB affects CSF concentrations of 5-Hydroxyindoleacetic acid (5-HIAA), homovanillic acid (HVA), and 3-Methoxy-4-hydroxyphenylglycol (MHPG), the monoamines of serotonin, dopamine, and norepinephrine. Another study conducted in Sweden showed that those born between January and April had higher levels of HVA and 5-HIAA in the CSF compared with those born between October and January [11].

Personalities are influenced by environmental and genetic factors [15]. The SOB also affects personalities. There are studies on the SOB and personality that have found an association between the SOB and neurosis, impulsivity, and sense of adventure [1]. Recent studies reported that there are associations between SOB and the Novelty Seeking (NS) and Reward Dependence (RD) dimensions of the Temperament and Character Inventory (TCI) [4, 12]. In adults, the SOB is associated with mortality and psychiatric and neurological disorders [5]. For example, Schizophrenics are 5–8% more likely to be born in winter or spring than in summer or fall. Studies have shown that it is associated with schizotypy in winter-born men [16]. This is probably because the male brain is vulnerable to prenatal stress [17]. The SOB was found to have an association with suicide attempts as well as psychiatric disorders. A study of suicides in Sweden Vasterbotten, between 1952 and 1993 showed that people born between October and January were more likely to commit suicide by poisoning or petrol gases, whereas those born between February and April were more likely to hang themselves [18].

Research on the relationship between the SOB and personality includes large cohort studies in Sweden and Finland [4, 15]. The Sweden study found that NS was higher in women born in Spring (between February and April) than winter (between October and January) [15]; the Finland study found that NS was higher in women born in Summer than in Winter [4]. These results can be summarized as higher NS in women born in spring or summer than in winter. Those born in winter had higher HVA in CSF and a lower NS than those born in spring [11]. There is also a study on the association between Dopamine receptor D2 (DRD2), a dopamine receptor gene, and personality that showed people with minor alleles of DRD2 had less brain dopamine activity [19, 20], NS and Persistence (PS) were higher in persons with minor alleles of DRD2 [21]. Another study examined the association between the DRD4 gene of the D4 dopamine receptor and personality and found that individuals with a 7-repeat allele (7R) in the DRD4 scored high on NS [21]. The effects of other gene alleles on the dopamine system have not been fully established.

Of note, these studies examined Caucasians in Northern Europe; there have been no large-scale studies conducted on the relationship between the SOB and personality in Asians. The TCI is a personality test developed based on a neurobiological model. Each temperament dimension in the TCI is closely related to molecular findings such as dopamine and serotonin [22]. Therefore, TCI may help in confirming the biological factors affecting the personality. Previous studies have confirmed the association with the SOB using TCI among Caucasians in Northern Europe. However, no large-scale studies have been conducted on the relationship between the SOB and personality in Asians. The present study investigates the relationship between these two factors using TCI in a large group of population in Korea.

## Materials and methods

### Study participants

This study used some of the data of the Regional University Specialization Project implemented at the Public Health Center of Kongju National University [23]. We obtained participation consents from the study participants after informing them of the confidentiality and the research use of their survey results. Of the 2962 survey respondents, 2656 were included, excluding the six who did not take the TCI test or answer important questions. The Institutional Review Board of Kongju National University approved this study.

### Temperament and Character Inventory (TCI)

The TCI is an inventory developed based on Cloninger’s psychobiological theory of temperament and personality. The inventory is designed to measure four dimensions of temperament—and three dimensions of character—Self-Directedness (SD), Cooperativeness (SO), and Self-Transcendence (ST). We used the Temperament and Character Inventory-Revised Short version (TCI-RS), 140-item form of the TCI that is psychometrically reliable and valid [24].

### The Patient Health Questionnaire-9 (PHQ-9)

The PHQ-9 consists of nine questions that were developed based on the nine item diagnostic criteria for a major depressive episode from the Diagnostic and Statistical Manual of Mental Disorders, 4th Edition (DSM-IV). It is used to assess depressive symptoms [25]. A total score ranging from 0 to 27. The validity and reliability of the Korean version of the PHQ-9 has been confirmed [26].

### Statistical analysis

We obtained descriptive statistics of general characteristics and characteristics of variables for data analysis. To confirm the difference in the TCI dimensions, we used the raw score to confirm the difference according to sex. The relationship between the SOB and TCI was analyzed using one-way analysis of variance (ANOVA), followed by A Bonferroni test as a post hoc analysis and comparisons of seasons of birth. For the NS dimension of TCI on which significant difference was found, linear regression analysis was performed to determine the influence of the SOB on its subscales. All data analyses were performed using SPSS 24.0 (IBM Inc., Armonk, NY, USA) with p<0.05 indicating statistical significance.

## Results

Of 2956 participants, 1458 (49.3%) were male and 1498 (50.7%) were female. The participants’ average age was 19.08 (± 1.09) years. Participants’ months of births were classified into four seasons: March through May into spring, June through August into summer, September through November into fall, and December through February into winter. The participant numbers for spring, summer, fall, and winter were 783, 647, 714 and 812, respectively (Table 1).

**Table 1:** General characteristics and research variables of the subjects (n = 2956).

Characteristics	Total
Sex	2956 (100%)
Male	1458 (49.3%)
Female	1498 (50.7%)
Age (years, means)	19.08 ± 1.090
Birth season	
Spring	783
Summer	647
Autumn	714
Winter	812
TCI, means	
NS	35.81 ± 9.26
HA	39.82 ± 10.97
RD	45.70 ± 8.69
P	40.74 ± 9.38
SD	45.50 ± 10.20
C	54.98 ± 9.19
ST	22.04 ± 9.62
SC	100.48 ± 15.93
PHQ-9, total	3.65 ± 3.39

Values are presented as either mean ± SD or n (%).

The ANOVA results on the SOB and TCI showed a statistically significant difference in the NS dimension. NS was lower in men born in spring than in fall or winter. The results of post hoc analysis also showed higher NS scores in men born in fall and winter than spring. For the relationship between the SOB and TCI in women, no statistically significant differences were found (Tables 2 and 3).

**Table 2:** The mean differences between TCI dimensions and seasons by gender.

dimensions	Male(n = 1458)						Female (n = 1498)					
	Spring	Summer	Autumn	Winter	F	*P*	Spring	Summer	Autumn	Winter	F	*P*
NS	34.78 ± 8.73	35.73 ± 9.36	36.73 ± 8.83	36.55 ± 8.94	3.839	0.009	35.72 ± 9.81	35.65 ± 9.34	35.47 ± 9.44	35.91 ± 9.56	0.139	0.937
HA	38.6 ± 10.36	38.98 ± 10.87	39.44 ± 10.30	39.2 ± 11.23	0.877	0.452	40.47 ± 11.44	40.80 ± 11.18	40.40 ± 10.94	40.93 ± 11.11	0.198	0.898
RD	34.76 ± 7.97	44.35 ± 8.55	44.34 ± 8.02	44.20 ± 8.57	0.421	0.738	47.97 ± 9.01	47.07 ± 8.83	46.79 ± 8.56	46.98 ± 8.75	1.331	0.263
P	42.06 ± 9.67	42.04 ± 9.20	40.97 ± 9.86	41.57 ± 9.04	1.036	0.376	40.60 ± 9.24	39.80 ± 9.68	40.12 ± 9.23	38.92 ± 8.79	2.316	0.074
SD	46.87 ± 10.12	46.15 ± 9.87	45.08 ± 9.73	45.63 ± 9.73	2.243	0.082	45.17 ± 10.38	45.14 ± 10.78	45.76 ± 11.09	44.27 ± 9.75	1.346	0.258
C	54.62 ± 9.07	54.52 ± 9.40	54.15 ± 9.18	45.00 ± 9.60	0.396	0.756	55.86 ± 9.08	55.14 ± 8.78	55.50 ± 9.08	55.93 ± 9.11	0.586	0.624
ST	20.80 ± 9.78	21.40 ± 9.53	22.17 ± 10.01	22.09 ± 10.00	1.645	0.177	22.97 ± 9.55	22.64 ± 8.92	21.44 ± 9.17	22.77 ± 9.70	2.038	0.107

NS, Novelty Seeking; HA, Harm Avoidance; RD, Reward Dependence; PS, Persistence; SD, Self-Directedness; CO, Cooperativeness; ST, Self-Transcendence.

**Table 3:** Multinomial logistic regression analysis of NS subscales on season of birth among men.

season	TCI	B	SE	Wald	OR (95% C.I.)	Sig.
summer	NS1	0.017	0.026	0.421	1.017 (0.966–1.071)	0.516
	NS2	0.001	0.030	0.001	1.001 (0.943–1.062)	0.976
	NS3	0.072	0.028	6.566	1.075 (1.017–1.136)	0.010
	NS4	-0.039	0.028	1.890	0.962 (0.910–1.017)	0.169
autumn	NS1	0.019	0.025	0.567	1.019 (0.970–1.071)	0.451
	NS2	0.001	0.029	0.002	1.001 (0.946–1.061)	0.960
	NS3	0.030	0.027	1.195	1.030 (0.977–1.086)	0.274
	NS4	0.055	0.027	4.118	1.056 (1.002–1.114)	0.042
winter	NS1	0.002	0.024	0.009	1.002 (0.956–1.051)	0.923
	NS2	0.006	0.028	0.046	1.006 (0.952–1.063)	0.831
	NS3	0.078	0.026	9.044	1.082 (1.028–1.138)	0.003
	NS4	0.006	0.026	0.055	1.006 (0.956–1.059)	0.814

NS1, Exploratory excitability; NS2, Impulsiveness; NS3, Extravagance; NS4, Disorderliness.

A multinomial logistic regression analysis of the SOB on four subscales of NS was performed to obtain more detailed information about the statistically significant relationship between the SOB and NS in men. The results showed significantly increased extravagance (NS3) in men born in summer and winter, and significantly higher disorderliness (NS4) in men born in autumn (Table 3).

## Discussion

This study investigated the association between the SOB and TCI in Korean college students. The one-way ANOVA and linear regression analysis on the SOB and TCI for the entire study participants yielded no statistically significant results. However, for men, NS was significantly lower in those born in spring than in fall or winter. Among NS dimension subscales, in our study, the difference between NS3 and NS4 was remarkable, and it was found that NS4 was significantly higher in males born in the fall. This is characterized by a little more freedom compared to other people. Men born in the summer and winter had higher levels of NS3. The lower NS3 and NS4 levels in men born in spring may suggest that they have a higher level of self-control and are more likely to be disciplined or follow rules [24].

It is known that the gender difference of TCI is higher in RD and HA in women than in men [27]. In our study, the variation according to sex and season was confirmed, there was no statistically significant correlation between the SOB and TCI among women, which is inconsistent with the large adult cohort studies in Sweden and Finland [4, 15]. In the Swedish study, women born in spring (between February and April) were higher on novelty seeking (NS), especially impulsiveness (NS2), than women born in winter (between October and January) [15]. In the Finnish cohort study, women born in winter were lower on NS than women born in summer [4]. In summary, women born in spring or winter were higher on novelty seeking (NS) than women born in summer, but these studies did not provide an explanation of why the SOB and personality had such relationship in women [28]. However, it can be deduced from the Russian study by Golimbet et al. that the genes involved in dopamine transmission include the catechol-O-methyltransferase (COMT) gene and the DRD4 gene. In the Russian study, NS was higher in participants with Met/Met genotype of COMT (catechol-O-methlytransferase) polymorphism than in participants with Val/Val or Val/Met genotypes of COMT polymorphism, although this result was only observed in women [29]. Many studies suggest an association between the COMD gene and DRD4 variant dopaminergic system genes and extraversion or NS only in women. Research on men suggests that COMT polymorphism is associated with interpersonal aggression and suicide [30, 31]. In animal studies, there was a gender difference in behavior associated with the COMT gene. Male mice with knock out DRD4 showed aggressive behavior, whereas female mice with knock out DRD4 showed emotional rather than behavioral reactions [32]. This result may suggest that the temperamental difference in men based on their SOB in the present study was likely influenced by the COMT gene. The reason why there is no difference in temperament according to season in women is may be because that the ratio of val/met polymorphism is relatively different in Koreans [33]. However, further research is needed to determine whether this difference was due to regional or racial characteristics.

A study conducted in the United States found that men born in winter scored higher on sensation seeking than men born in other seasons [34]. Sensation seeking is a personality trait that involves seeking new experiences, thrills, and adventures or impulsive behavior, and it is similar to TCI’s NS. The similarity between that study and the present study is that the study participants were college students (mean age of 19.33 and 19.08, respectively); the participants’ race was the difference between the studies as the majority (44%) of the U.S. study’s participants were European descent [15, 35, 36]. In another cohort study, NS was elevated in women born in winter, although this gender-specific outcome is inconsistent with the findings of the present study. According to a previous study, age is a factor that influences personality traits. Sensation seeking is affected by age, specifically higher in younger people [34]. Between birth and around 20 years of age, sensation seeking increases [37, 38]; it peaks between 26 and 30 years old, and then declines. Gender also affects personality. Studies have shown that men are more sensation seeking than women. While the ability to accept a new experience was the same between men and women, men’s sensation seeking was more proactive [38].

There are several hypotheses about the mechanism by which SOB affects personality traits. The first is the hypothesis that photoperiod during pregnancy or the neonatal period associated with the SOB affects dopamine turnover. The SOB affects the monoaminergic system of the neonatal brain. The brain monoamine turnover rate is related to personality [39–41]. The HVA level in the CSF is proportional to the amount of dopamine activity in the brain [42, 43], and there is research showing that as dopamine activity increases and dopamine turnover increases, NS decreases [19, 20]. High NS is known to be associated with low dopamine action [44]. The men born in winter have relatively high NS that is associated with high dopamine activity, which can be considered as a link to some psychopathology. The second hypothesis postulates that personality is related to the circadian rhythm. Melatonin and dopamine inhibit each other. That is, at night, as melatonin increases, dopamine decreases; a reversal of these levels occurs during the day [4]. Natale et al. found that those born in winter were more likely to be a morning person than those born in summer, and suggested that the dopamine levels of those born in winter are higher than those born in summer [45]. Interestingly, Caci et al. found that morning people are lower on the NS dimension [46]. The melatonin rhythms of the mother and the child are related, and the mother’s melatonin rhythm changes with the season during pregnancy [47]. Hofman et al. found in their study of autopsies, melatonin levels in the pineal gland were lower at night in bodies examined in winter (between October and March) than bodies examined in months with longer daytime [48].

The limitations of this study are as follows. First, as this study’s participants were students of a Korean university, future research needs to include a wider range of participants. Second, the study employed the Korean conventional method of classifying season into four seasons which is different from previous studies’ classification method. Third, the objectivity of interpretation may be limited due to it being a cross-sectional study with only a self-report test. Fourth, it is difficult to know the direction of the interaction between SOB and TCI due to the nature of cross-sectional studies, and we were unable to determine what clinical outcomes could result from this interaction.

Nevertheless, this study has significance as the first large-scale Asian study on the relationship between SOB and TCI. Previous studies mostly analyzed individuals from western countries. Especially, the Swedish and Finish studies conducted in countries with similarities in genetic and environmental factors such as race, latitude, solar radiation, and temperature. Temperaments are traits, and can be explained by the phenotype of learning and memory made during growth [22]. It reflects complex genetics and developmental processes. In the future, follow-up studies need to examine the association between SOB, personality traits, and related psychiatric disorders. Investigation of this association will help further the understanding of the pathology and treatment of psychiatric disorders including mood disorders.

## Conclusions

This study investigated the relationship between SOB and TCI. Although no significant differences in personality in women or men were found, NS, a temperament dimension, was higher in men born in fall or winter than in spring. This finding is consistent with the previous study findings on male adolescents and young adults. Taken together with previous studies, winter births in humans may be associated with dopamine vulnerability, which is likely to be the effect of photoperiod during the perinatal period. Development of the dopamine system should be investigated to understand its association with the intrauterine environment or the maternal circadian system. Follow-up research on the topic will be needed.

## Supporting information

S1 Table The mean differences between TCI NS subdimensions and seasons in male. (DOCX)
