# Supplementary material for: Is personality linked to season of birth?
Source: PLoS One. 2021 Jun 25;16(6):e0253815. doi: 10.1371/journal.pone.0253815 (PMC8232405; doi:10.1371/journal.pone.0253815)
Supplement: S1 Table — (DOCX) [file pone.0253815.s001.docx]

S1 Table. The mean differences between TCI NS subdimensions and seasons in male

|  | spring | summer | autumn | winter | F | *P* |
| --- | --- | --- | --- | --- | --- | --- |
| NS1 | 10.61 ± 3.12 | 10.84 ± 3.30 | 10.96 ± 3.32 | 10.80 ± 3.25 | 0.751 | 0.522 |
| NS2 | 8.37 ± 3.14 | 8.59 ± 3.32 | 8.82 ± 3.19 | 8.80 ± 3.23 | 1.695 | 0.166 |
| NS3 | 7.44 ± 3.08 | 8.02 ± 3.22 | 7.93 ± 3.17 | 8.23 ± 3.01 | 4.695 | 0.003 |
| NS4 | 8.38 ± 3.10 | 8.29 ± 2.99 | 9.03 ± 3.26 | 8.72 ± 2.94 | 4.238 | 0.005 |
